# Gasotransmitters in Action: Nitric Oxide-Ethylene Crosstalk during Plant Growth and Abiotic Stress Responses

**DOI:** 10.3390/antiox8060167

**Published:** 2019-06-08

**Authors:** Zsuzsanna Kolbert, Gábor Feigl, Luciano Freschi, Péter Poór

**Affiliations:** 1 Department of Plant Biology, University of Szeged, 6726 Szeged, Hungary; feigl@bio.u-szeged.hu (G.F.); poorpeti@bio.u-szeged.hu (P.P.); 2 Laboratory of Plant Physiology and Biochemistry, Department of Botany, University of Sao Paulo, Sao Paulo 05422-970, Brazil; freschi@usp.br

**Keywords:** abiotic stress, ethylene, growth and development, nitric oxide

## Abstract

Since their first description as atmospheric gases, it turned out that both nitric oxide (NO) and ethylene (ET) are multifunctional plant signals. ET and polyamines (PAs) use the same precursor for their synthesis, and NO can be produced from PA oxidation. Therefore, an indirect metabolic link between NO and ET synthesis can be considered. NO signal is perceived primarily through S-nitrosation without the involvement of a specific receptor, while ET signal is sensed by a well-characterized receptor complex. Both NO and ET are synthetized by plants at various developmental stages (e.g., seeds, fruits) and as a response to numerous environmental factors (e.g., heat, heavy metals) and they mutually regulate each other’s levels. Most of the growth and developmental processes (e.g., fruit ripening, de-etiolation) are regulated by NO–ET antagonism, while in abiotic stress responses, both antagonistic (e.g., dark-induced stomatal opening, cadmium-induced cell death) and synergistic (e.g., UV-B-induced stomatal closure, iron deficiency-induced expression of iron acquisition genes) NO–ET interplays have been revealed. Despite the numerous pieces of experimental evidence revealing NO–ET relationships in plants, the picture is far from complete. Understanding the mechanisms of NO–ET interactions may contribute to the increment of yield and intensification of stress tolerance of crop plants in changing environments.

## 1. Introduction

### 1.1. Biochemistry, Synthesis, Storage, and Transport of NO and ET in Higher Plants

Nitric oxide (NO, N=O) is one of the smallest diatomic gas (30.006 g mol^−1^), containing a double bond between the N and the O atoms, with active redox character due to the presence of one unpaired electron [1]. Ethylene (ET, C_2_H_4_, CH_2_=CH_2_) is also a small and simple gaseous molecule (28.054 g mol^−1^), containing two C atoms with a double bond and no radical character or redox nature. Both NO and ET are small, uncharged and lipophilic molecules; therefore, they are capable of diffusing across membranes and travel easily from cell to cell.

Early reports described NO as an atmospheric gas influencing growth of aerial plant parts [2,3,4,5]. In the case of ET, the same conclusion was drawn in the early studies; however, these observations were made much earlier compared to NO (in 1858 and 1901, [6]). Until the 1960s, ET was exclusively examined as a promoter of fruit ripening; however, intense research on ET influence on other growth responses was subsequently started [7,8,9,10]. Therefore, ET research preceded NO research since the second phase of NO-related plant studies only began in the 1990s.

**Figure 1:**
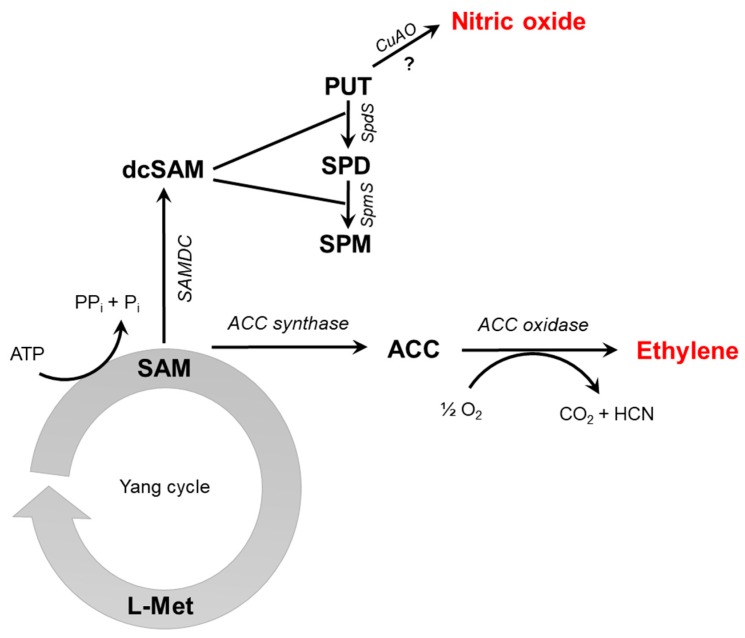
Schematic overview of ethylene synthesis intermediates (S-adenosyl-methionine, SAM; 1-aminocyclopropane-1-carboxylic acid, ACC) and enzymes (ACC synthase, ACC oxidase). The formation of nitric oxide through putrescine (PUT) oxidation by copper-amine-oxidase (CuAO) is also depicted; however, the question mark indicates that this mechanism requires further experimental evidence. Additional abbreviations: L-Met, L-methionine; SAMDC, SAM decarboxylase; dcSAM, decarboxylated SAM, SPD, spermidine; SpdS, spermidine synthase; SPM, spermine; SpmS, spermine synthase.

In higher plants, the production of NO is tightly connected to plant nitrate assimilation [11] where reductive reactions lead to NO formation. Nitrate reductase (NR) was shown to directly reduce nitrite to NO; however, its NO-producing activity is only 1% of its nitrate-reducing activity in vitro [12]. An indirect role NR in NO production has also recently emerged, based on the NR-mediated transfer of electrons from NAD(P)H to the NO-forming nitrite reductase (NOFNiR) which in turn catalyzes the in vitro and in vivo reduction of nitrite to NO [13]. However, the significance of NR-NOFNiR system in NO synthesis during stress responses still remains to be elucidated. Besides NR, root-specific nitrite:NO reductase (NiNOR) [14] were shown to produce NO using nitrite as a substrate. Non-enzymatic NO synthesis has also been demonstrated under specific conditions, such as in the apoplast of barley seed aleurone layer where nitrite is reduced at acidic pH in the presence of ascorbate leading to the formation of NO [15]. Oxidation of reduced nitrogen compounds (L-arginine, polyamines or hydroxylamine) is an additional way to release NO by plant tissues. Using biochemical approaches, L-arginine-dependent NO formation was detected in different plant species [16]; although the gene for mammalian-like nitric oxide synthase (NOS) enzyme has not been identified in land plants ever since [17]. Therefore, Corpas and Barroso [18] argue the hypothesis that L-arginine-dependent NOS-like activity in higher plants could be the result of cooperation between discrete proteins. In contrast to higher plants, in algae like *Ostreococcus tauri* and *Synechococcus* PCC 7335 NOS enzymes functionally and structurally similar to mammalian NOS have been characterized [19,20]. Also, polyamines (PAs) are good candidates for oxidative NO release; however, the mechanism is still unclear. Copper-amine oxidase1 (CuAO1) was found to be involved in PA-induced NO formation as *cuao1-1* and *cuao1-2* mutants showed prevented PA-induced NO formation [21]. Later, Groß et al. [22] found that low NO level of *cuao8 Arabidopsis* is associated with increased arginase activity, which can contribute to lower NO production due to poor availability of arginine. An important precursor of PA synthesis is S-adenosylmethionine (SAM) [23], which is also the substrate for ET biosynthesis. In the first step, SAM is converted to 1-aminocyclopropane-1-carboxylic acid (ACC) by the enzyme ACC synthase (ACS). The other product of ACS activity is methylthioadenosine, which is recycled to methionine (Met) in the Yang cycle to maintain intracellular Met level. The ACC is then oxidized by ACC oxidase (ACO) in the presence of Fe(II) cofactor, oxygen and ascorbate resulting in the evolution of ET, CO_2_, and HCN. Therefore, compared to other plant hormones, the biochemical pathway of ET synthesis in plants seems to be relatively simple. Both ACS and ACC are encoded by multigene families and the mRNA levels and activity of each isoenzyme are differently regulated by endogenous (e.g., auxin, NO) and environmental factors (e.g., flooding, drought, pathogen attack) [24]. The relationship between the biosynthetic pathways of NO and ET is depicted in Figure 1.

As discussed above, it is widely accepted that NO can be synthesized in higher plants by oxidative and reductive mechanisms involving enzymes and also non-enzymatic processes. The substrates for oxidative pathways include aminoacids (L-arginine) or compounds containing two or more aminoacids (polyamines). The copper amine oxidase-catalysed degradation of PAs leads to NO production. Due to the common precursor of PA and ET synthesis (SAM), the NO and ET synthesis are linked via PA metabolism.

Both NO biosynthesis and removal are critically important to regulate steady-state NO levels in plant cells. In the presence of molecular oxygen, NO forms nitrite and nitrate, and it interacts with superoxide anion to form peroxynitrite (ONOO^−^) [25]. Moreover, NO may also react with proteins, like hemoglobin (Hb), and this interaction facilitates its oxidation to nitrate [26,27]. Another possible mechanism for NO removal involves NR enzyme which can transfer electrons to the truncated hemoglobin THB1 catalyzing the conversion of NO into nitrate by its dioxygenase activity [11,28]. NO can initiate S-nitrosation reactions with thiol (SH)-containing proteins and peptides resulting in the formation of low-molecular-weight S-nitrosothiols such as S-nitrosocysteine (CysNO) or S-nitrosoglutathione (GSNO) [29,30]. The *S*-nitrosothiols liberate NO and participate in transnitrosation or *S*-thiolation [29,31]. The most abundant S-nitrosothiol is GSNO which can non-enzymatically generate NO or be reduced by the enzyme *S*-nitrosoglutathione reductase (GSNOR), yielding oxidized glutathione (GSSG) and ammonia (NH_3_) [32]. Besides being an intracellular NO reservoir, GSNO may also be transported between cells, tissues, and organs implementing long-distance transport of NO signal [33].

In the case of ET, the inactivation by oxidation is not physiologically relevant in regulating steady-state ET levels due to its fast diffusion from tissues. Short distance movement of ET occurs via diffusion from cells into intercellular gas spaces and into the environment, while its long-distance transport from is proved to be ACC [34].

### 1.2. Perception and Transduction of NO and ET Signals

Due to the lack of known specific receptors, perception and transduction of NO signal are believed to rely on posttranslational modifications (PTMs) such as S-nitrosation, tyrosine nitration and metal nitrosylation of target proteins [35]. S-nitrosation is a reversible covalent reaction affecting cysteine thiol groups and consequently modifying protein activity, localization or interactions. This PTM is catalyzed by higher oxides of NO or nitrosonium cation (NO^+^), metal-NO complexes, and low molecular weight S-nitrosothiols (CysNO) or GSNO [36]. According to the most comprehensive dataset of S-nitrosated proteins, 1,195 endogenously S-nitrosated peptides belonging to 926 proteins can be found in the *Arabidopsis* proteome [37].

Moreover, NO can influence protein activity indirectly through the formation of ONOO^−^ in a reaction with superoxide. Peroxynitrite formation leads to protein tyrosine nitration (PTN). PTN is an irreversible two-step PTM during which a nitro group (-NO_2_) binds to the aromatic ring of tyrosine (Tyr) in the *ortho* position resulting in the formation of 3-nitrotyrosine [38]. In plant cells, PTN inhibits the activity of the particular enzyme protein as reviewed in [39]. Tyrosine nitration may either prevent or induce the tyrosine phosphorylation; thus, it can influence cell signalling [39]. In a comprehensive study, 127 nitrated proteins were identified in wild-type, control *Arabidopsis thaliana* [40] indicating that plants possess a physiological nitroproteome [39]. Nitric oxide can also bind to transition metal ions like iron (Fe^2+^ or Fe^3+^), copper (Cu^2+^) or zinc (Zn^2+^) in metalloproteins to form metal-nitrosyl complexes [41]. The biological significance of metal nitrosylation; however, needs to be further analysed.

Contrary to NO signalling, ET perception and signalling involve the function of specific receptors. ET is perceived by a receptor complex located in the endoplasic reticulum membrane, which is related to the histidine protein kinase receptors of the two-component signalling system found in prokaryotes. In *Arabidopsis*, five ET receptors have been identified (ETHYLENE RECEPTOR1 and 2, ETR1, ETR2; ETHYLENE RESPONSE SENSOR1 and 2, ERS1, ERS2; ETHYLENE INSENSITIVE4, EIN4). The N-terminal transmembrane domain of the receptors binds ET in the presence of a copper co-factor. Without ET, the receptors activate a serine/threonine protein kinase CONSTITUTIVE RESPONSE1 (CTR1), which in turn negatively regulates the downstream ethylene response pathway, possibly through a MAP-kinase cascade. Once ET binding takes place, the receptors become inactive, resulting in deactivation of CTR1, which in turn causes ETHYLENE INSENSITIVE2 (EIN2) to function as a positive regulator of ET signalling. EIN2 contains the N-terminal hydrophobic domain and the hydrophilic C-end, and positively regulates nucleus-located ETHYLENE INSENSITIVE3 (EIN3) transcription factors (TFs). EIN3 binds to the promoter element of ETHYLENE RESPONSE FACTOR (ERF1) gene and activates its transcription in an ET-dependent manner. Transcription factors like ERF1 and other ethylene-response-element binding proteins (EREBPs) can interact with the GCC box in the promoter of target genes and activate downstream ET responses [24]. Evidence indicates that group VII of the ERF/AP2 transcription factor family (i.e., ERFVIIs) may be implicated as sensors of NO availability during early seedling development (i.e., seed germination and hypocotyl elongation) and stomatal closure [42]. It has been shown that ERFVII-dependent NO sensing relies on the specific oxidation of the C2 residue of these TFs, leading to their arginylation and polyubiquitylation and subsequent proteasomal degradation via the N-end rule proteolytic pathway. Whether the NO-dependent modulation of ERFVII stability is a central mechanism controlling other NO–ET interactions throughout the plant life cycle remains to be investigated.

Based on the above, NO and ET signal transduction mechanisms in plant cells are fundamentally different. NO has no specific receptor; thus the NO signal is perceived at the proteome level, and it leads to signal transduction and gene expression response primarily via specific PTMs. Contrary to NO, ET signal is sensed by a well-characterized, specific receptor complex, having the negative regulation in its signalling cascade as distinctive characteristic.

ET is considered to be a classical plant hormone because it is detected through specific receptors and acts at low concentrations (0.01 to 1.0 ppm) [43]. On the other hand, since the NO signal is not perceived by specific receptors and the range of its effective concentration is apparently higher than those of the classical phytohormones, we presently do not consider NO as a plant hormone, but instead it is regarded as a non-traditional growth regulator that acts in interaction with traditional phytohormones, including ET, during plant growth and development. The further sections of this review intend to discuss these wide-scale interactions between NO and ET in plant growth and development under either optimal or stressful conditions.

## 2. NO–ET Interplay during Plant Development

**Figure 2:**
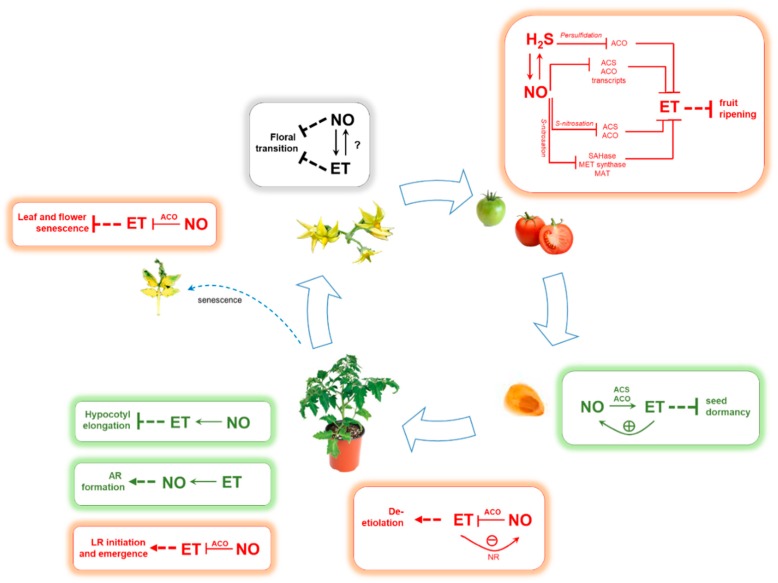
Nitric oxide (NO)-ethylene (ET) interactions during plant growth and development. Synergistic interplay was indicated in green, while antagonism was indicated in red. Abbreviations: ACS, 1-aminocyclopropane-1-carboxylic acid synthase, ACO, aminocyclopropane-1-carboxylic acid oxidase; SAHase, adenosyl homocysteinase; MET synthetase, methionine synthetase; MAT, methionine adenosyltransferase; LR, lateral root; AR, adventitious root.

In conjunction with other regulatory signals, NO and ET are known to influence a vast array of developmental processes during the plant life cycle. Over the years, these two gaseous signals have been demonstrated to closely interact to control key biological processes in early plant development as well as in vegetative growth, fruit ripening and leaf senescence (Figure 2).

### 2.1. NO–ET Crosstalk during Seed Germination

The ultimate goal of zygotic embryogenesis is to produce a viable seed, which has the capacity to germinate. Environmental factors, including temperature, water availability and day length, play a crucial role in the maintenance and break of seed dormancy, whose main function is to prevent germination when circumstances are not favouring seedling survival [44]. Beyond the effects of environmental factors, seed dormancy is also controlled by numerous endogenous factors; primarily associated with changes in the balance of the hormones abscisic acid (ABA) and gibberellins (GAs) [45]. Due to its crosstalk with ABA and GA, ET is also involved in seed development [46], having a well-known role in the removal of seed dormancy and promotion of germination [47,48]. Similarly, NO also induces germination in numerous plant species [15,49,50] and the aleurone layer seems to be relevant in NO generation and signalling [51].

In general, a synergistic link seems to exist between NO and ET during seed germination at various levels [52]. Besides counteracting ABA-imposed seed dormancy [53] and facilitating the onset of GA-stimulated germination [51], NO has also been shown to promote the breaking of apple embryonic dormancy by stimulating ET emission [54]. The NO-triggered release of apple embryo dormancy correlated with enhanced ET production and was abolished when ET synthesis was inhibited [54]. In line with this, subsequent studies demonstrated that the NO-triggered increment in ET production in germinating apple embryos was associated with the stimulation of both ACS and ACO activities [55]. Altogether, these findings indicate the involvement of endogenous ET production in NO-triggered dormancy breaking.

In the case of *Amaranthus retroflexus* seeds, the germination was induced by exogenous NO, and this effect was preceded by increased ET production, indicating that the NO-induced dormancy breaking is also ET-dependent. On the other hand, ET-induced seed germination has also been shown to require NO presence. Moreover, both NO and ET-induced seed germination were associated with the activation of the cell cycle before radicle emergence [56].

### 2.2. NO–ET Interplay during Vegetative Growth

As a multifunctional plant hormone, ET can either stimulate or inhibit plant growth depending on its concentration, on the duration of the application and the plant species [57]. According to many reports, the interaction between NO and ET tends to be more antagonistic during plant vegetative growth; however, it largely depends on the exact process or organ investigated [58].

During light-induced greening and chloroplast differentiation, either endogenously produced or exogenously applied NO was found to promote these de-etiolation-related processes by inhibiting ACO activity and consequently repressing ET biosynthesis (and inducing auxin synthesis) in tomato (*Solanum lycopersicum*) cotyledons. Furthermore, pharmacological and genetic approaches revealed that higher ET levels inhibited NO formation and total NR activity, suggesting a mutual negative interaction between ET and NO signalling during de-etiolation of tomato seedlings [59].

Transcriptomic and genetic evidence based on *Arabidopsis* sensitivity to NO in hypocotyl shortening further indicated that NO and ET signalling are connected during early seedling development [60]. Remarkably, the ethylene-insensitive mutants *etr1-3* and *ein2-5* mutants were completely insensitive to exogenous NO-induced hypocotyl shortening, thereby providing further support to the involvement of ET signalling in NO sensing during early seedling development.

Few reports describe the ET-NO interaction events during root development. Ethylene negatively regulates lateral root (LR) initiation, growth and elongation in *Arabidopsis* [61,62], while NO was found to induce LR growth in tomato [63]. Recently, it was observed that NO-induced LR initiation possibly relies on the inhibition of ACO in sunflower seedlings. Authors hypothesized several ways of NO action: (1) NO may modify the ferrous site of ACO leading to its inhibition, (2) NO may inhibit the expression of *ACO* genes, and/or (3) NO may modify the expression of transcription factors leading to reduced *ACO* gene expression [64]. Moreover, in selenium (Se)-stressed root system of *Arabidopsis*, the increment in LR emergence was accompanied by elevated ET levels which were shown to inhibit NO production. Exogenous NO (GSNO) decreased ET levels suggesting a mutually negative interplay between NO and ET during Se-induced LR emergence [65].

Both NO and ET also play a role in the induction of adventitious root (AR) development [66,67,68]. As a rare exception investigating NO–ET crosstalk, Jin et al. [69] reported that exogenous application of ethylene-releasing compound 2-chloroethylene phosphonic acid (ethephon) induced both AR formation and NO production in marigold (*Tagetes erecta*) whereas scavenging of NO (by cPTIO) significantly inhibited AR-inducing capacity of ethephon. The authors concluded that the AR-inducing effect of ET relies on endogenous NO generation in marigold roots [69] suggesting a synergism between ET and NO in this process. 

At the cellular level, ET-NO interaction was investigated in *Arabidopsis* cell culture [70]. Both NO and ET were produced by wild-type (WT) cell culture in the period of active cell division while *ein2* cells failed to produce ET but emitted increased NO levels. Moreover, low concentrations of NO donor (SNP) stimulated G1/S phase transition and reduced ET levels in WT *Arabidopsis* cells, suggesting an antagonistic relationship between ET and NO levels during cell cycle progression. Higher NO donor concentrations restrained S phase transition indicating the concentration-dependent effect of NO on cell division. Interestingly, *ein2* cells were insensitive to a low dosage of NO donor suggesting that the effect of NO on cell division involves EIN2-associated ET signalling.

### 2.3. NO–ET Interaction during Reproductive Growth

Although both NO and ET have been reported to repress floral transition in *Arabidopsis* [71,72] whether these signalling molecules crosstalk to integrate external and internal signals into the floral decision remains to be investigated. NO is actively produced during floral development until anthesis [73], and *Arabidopsis* mutants with altered NO levels usually display limited fertility [74]. A chemotropic role has been attributed to NO during pollen tube navigation and ovule targeting [75], which may be one of the reasons behind the low fertility, reduced silique size and limited seed production in *Arabidopsis* mutants with disturbed NO levels such as the *nitric oxide overexpression 1* (*nox1*) and the *AtGSNOR1* loss-of-function *atgsnor1-3* [72,74]. Among fleshy fruits, reduced fruit size associated to increased endogenous NO levels has also been reported for the tomato mutant *short root* (*shr*), which also displayed marked reduction in flower size and fertility [76]. Though evidence indicates that ET influences plant sexual reproduction via multiple mechanisms, including the regulation of pollen tube growth [77], ovule lifespan [78] and fruit set [79], some potential NO–ET crosstalk during these responses remains elusive.

Since the seminal studies carried out by Leshem’s group [80,81], NO has emerged as a potent molecule capable of extending shelf-life of postharvest fruits of several important crop species ([82] and references therein), acting as an antagonist of ET in many of these cases. Ripening-associated processes typically promoted by ET, such as cell wall softening, chlorophyll degradation and synthesis of new pigments, are inhibited or delayed by NO treatment [83], thereby leading to an extension in postharvest fruit shelf life ([84] and references therein). Biochemical routes leading to the synthesis of important fruit nutritional compounds, such as carotenoids, flavonoids, and ascorbate, also are under strict regulation by the NO and ET interplay. For example, carotenoid synthesis and ascorbic acid degradation are both promoted by ET [85,86] and inhibited by NO [87,88] during fruit ripening. Therefore, the final nutritional attributes of fruits will ultimately depend on the combined influence of these two gasotransmitters during the ripening and post-ripening phases. Moreover, NO treatment has also been shown to delay or ameliorate the development of physiological disorders and disease incidence during postharvest storage, particularly when combined with low-temperature conditions [84,89].

In contrast with the wealth of information about ET biosynthesis and signalling during fruit ripening, data on NO metabolism in ripening fruits remain relatively scarce. By employing non-invasive photoacoustic spectrometry, Leshem and Pinchasov [81] revealed an opposite trend between NO and ET emission rates during both climacteric and non-climacteric ripening, with NO and ET predominating in the green and ripe stage, respectively. In agreement, endogenous ACC and NO levels displayed an inverse correlation during the abscission of mature olive (*Olea europaea*) fruits [90]. In pepper (*Capsicum annuum*), the transition from green to red stage is associated with a decline in NO levels and the accumulation of both nitrosated and nitrated proteins, which also coincided with the reduction in GSNOR activity [91,92]. Intriguingly, although fruits of NO-hyperaccumulator tomato mutant *shr* displayed slower ripening compared to WT counterparts, no significant differences in ET emission were observed between *shr* and WT fruits at the climacteric phase [76]. 

Climacteric fruits, which strongly relies on ET signalling for ripening initiation, predominate among the representative examples of NO–ET antagonism during ripening, including several major fleshy fruit crops such as apple [93], banana (*Musa spp.*, [94]), tomato [87], papaya (*Carica papaya*, [95]), peach (*Prunus persica*, [96]) and mango (*Mangifera indica*, [97]). In these fruits, the climacteric peak of ET production is either reduced or delayed in response to NO treatment [87,94], a response frequently associated with NO-triggered reductions in transcript abundance and activity of key ET biosynthetic enzymes, particularly ACS and ACO [97]. In some cases, the lower ACO and ACS activities detected in NO-treated fruits were at least partially explained by the decline in their encoding transcripts [87,94]. However, the direct inhibition of ACS and ACO via NO-mediated PTM cannot be ruled out [58].

NO-mediated PTM events are assumed to affect SAM turnover in plants as the methylmethionine cycle enzymes adenosyl homocysteinase (SAHase), methionine synthase (MET synthase) and methionine adenosyltransferase (MAT) have all been identified as common targets of S-nitrosation in GSNO-treated *Arabidopsis* leaf extracts [98]. Further support for MET synthase as a target of S-nitrosation was provided when the biotin-switch technique was conducted in GSNO-treated leaf extracts of other species, such as *Kalanchoë pinnata* and *Brassica juncea* [99,100]. Moreover, Lindermayr et al. [101] have demonstrated that *Arabidopsis* MAT1 activity is inhibited by S-nitrosation at Cys-114 under in vitro conditions.

It is also possible to conceive more complex scenarios where NO inhibits fruit ET biosynthesis via intermediary steps. NO is a central player in plant redox metabolism and homeostasis [102], and the activity of antioxidant enzymes such as catalase (CAT) and ascorbate peroxidase (APX) can be modulated via S-nitrosation during fruit ripening [103]. As both fruit ripening and ET biosynthesis are influenced by redox signalling [83], an indirect action of NO on ripening via changes on fruit redox state remains an unexplored possibility. Moreover, hydrogen sulfide (H_2_S) has been recently proposed as part of the NO–ET crosstalk in ripening fruits, with NO and H_2_S synergistically interacting to inhibit ET production during fruit ripening [82,103,104]. Evidence indicates that H_2_S can act either upstream or downstream NO depending on the physiological process considered [103], and H_2_S has been proposed to reduce ET production in tomato plants by inhibiting ACO activity via persulfidation of Cys-60 [77]. Therefore, a complex interplay between NO-H_2_S-ET may be involved in fruit ripening, and this emerging interplay certainly deserves further investigation.

### 2.4. NO–ET Interaction during Senescence

ET is largely accepted as a key promoter of leaf, flower and fruit senescence, whereas NO plays an opposite role [58]. The senescence-promoting role played by ET is confirmed by the premature senescence symptoms such as leaf yellowing, necrosis and abscission triggered in many species upon ET exposure [57]. The rise in endogenous ET production during both natural and stress-induced senescence as well as the altered senescence phenotype of many ET biosynthesis and signalling mutants also support the promotive effect of ET on leaf senescence [57]. Similarly, climacteric ET production is triggered in many flowers soon after pollination, leading to wilting, fading and abscission, whereas the treatment of flowers with inhibitors of ET biosynthesis or action frequently leads to delayed senescence ([105] and references therein).

In contrast, NO fumigation or the exposure of the plants to conditions that promote endogenous NO levels (e.g., high nitrate) usually delay leaf senescence [105], and treatments of flowers with NO donors have long been described to extend their vase life [106]. Moreover, leaf senescence is accelerated whenever endogenous NO content is reduced, such as in NO-deficient mutants [107] and transgenic plants overexpressing a NO-degrading dioxygenase (NOD) gene [108]. Young plant tissues usually display higher NO levels, which progressively decrease as plant organs mature [109,110], with ET displaying an opposite trend [111]. Therefore, plant ET and NO metabolisms seem to be inversely influenced by tissue aging.

Little is known about the mechanisms behind the NO–ET crosstalk regulating leaf and flower senescence. In line with the apparent antagonistic relationship between these gasotransmitters during senescence, the leaf senescence-like phenotype of NOD-expressing *Arabidopsis* transgenic plants coincided with increased expression of *AtACS6* [108], which is one of *ACS* genes responsible for the enhanced ET production during leaf senescence in this species [112]. More recently, Niu and Guo [113] provided genetic evidence based on the use of both the NO-deficient mutant *Atnoa1* and ethylene-insensitive mutant *ein2-1* suggesting that the NO action during dark-induced senescence in *Arabidopsis* involves ethylene insensitive 2 (EIN2), which a positive regulator of ET signalling and leaf senescence [114]. The premature senescence observed in the NO-deficient *Atnoa1* mutant was attenuated by mutations in EIN2, and the dark-triggered induction of senescence marker genes and thylakoid membrane integrity loss was significantly delayed in the *Atnoa1 ein2-1* double mutant [113].

In cut flowers, the increment of vase life upon NO treatment also seems to involve an antagonistic influence of this free radical on the ET biosynthetic pathway. Besides counteracting the ET promotive effects on flower senescence in several species [106], exogenous NO has been shown to reduce ACO activity and ET emission in cut rose [115]. Therefore, similarly to observed in fruit ripening, exogenous NO seems to negatively influence ET biosynthesis in postharvest cut flowers. Whether NO action is also relevant during the natural senescence of flowers triggered by pollination or ageing remains to be investigated.

## 3. NO–ET Interplay in Abiotic Stress Responses

**Figure 3:**
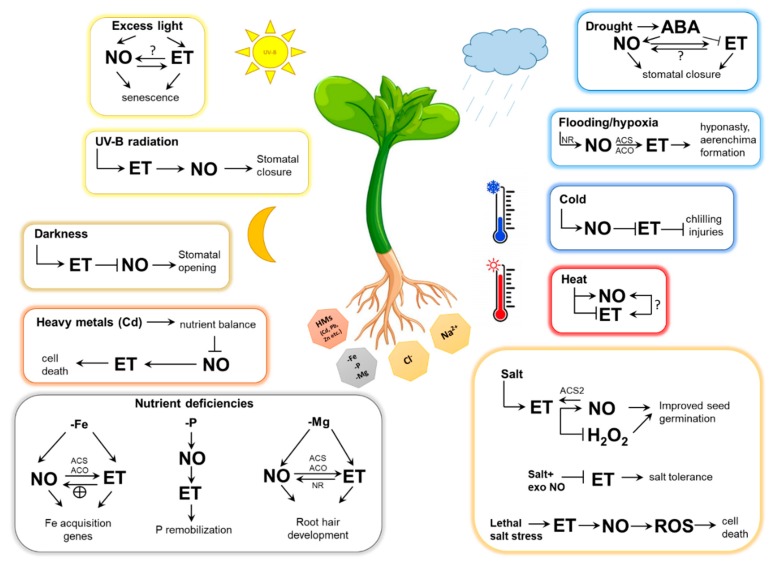
Schematic representation of nitric oxide (NO)-ethylene (ET) interplay during abiotic stress responses in plants. Abbreviations: ABA, abscisic acid; ACS, 1-aminocyclopropane-1-carboxylic acid synthase, ACO, aminocyclopropane-1-carboxylic acid oxidase, NR, nitrate reductase; H_2_O_2_, hydrogen peroxide; ROS, reactive oxygen species; exo NO, exogenous NO; HMs, heavy metals; -Fe, iron deficiency; -P, phosphorus deficiency; -Mg, magnesium deficiency.

Environmental stresses (e.g., excess light, cold, heat, salt, drought, flooding, nutrient deficiencies, heavy metals) are relevant determinants of plant physiological processes. Plant responses to these abiotic stresses are regulated by crosstalk between multiple signal molecules, including NO and ET (Figure 3).

### 3.1. NO–ET Crosstalk during Light Stress Responses

Light not only provides energy for photosynthesis but also represents a crucial environmental signal responsible for adjusting plant growth, development, and reproduction. Processes as diverse as seed germination, seedling de-etiolation, phototropism, flowering, fruit pigmentation, and entrainment of circadian rhythms are intrinsically regulated by light stimuli [116]. However, excess light intensity and UV-B-enrichment can negatively impact photosynthetic efficiency by inducing photoinhibition, which is linked with excessive reactive oxygen species (ROS) generation [108,117,118]. In contrast, photosynthesis is limited or halted when light is absent or below optimal levels. Either way (excessive or insufficient light) can trigger changes in both NO and ET metabolism, as well as in other phytohormones [119].

Significantly elevated NO and ET emissions upon light stress have been found in *Arabidopsis* [119]. High light intensity (555 and 1500 µmol m^−2^ s^−1^) and short time of the light exposure (2-4-6 h) significantly increased NO emission in *Arabidopsis* shoot, but it was reduced under darkness after 12 h [119]. Moreover, high light exposure (500 µmol m^−2^ s^−1^) compared to the control illumination (70 µmol m^−2^ s^−1^) resulted in the attenuation of NOD activity and induced senescence in *Arabidopsis thaliana* by stimulating NR activity and enhancing NO emission [108]. Therefore, NO–ET interplay seems to positively influence high light-induced senescence but the interaction between the two gasotransmitters in light-stressed plants requires further attention.

Similar to high light, UV-B radiation also results in significant ET and NO production in numerous plant species and organs [120,121]. NO–ET crosstalk in UV-B-stressed plants has been firstly evidenced by using chemical modulators [122]. Scavenging of UV-B-induced NO production using PTIO resulted in the repression of UV-B-triggered ET emission. At the same time, exogenous NO donor treatments (SNP) promoted the UV-B-induced ET accumulation in the seedlings. Authors concluded that NO could promote ET accumulation under UV-B stress [122]. Also, stomatal closure induced by UV-B radiation has been reported in *Vicia faba,* which was promoted by NO accumulation in guard cells after the ET evolution peak [123]. Both UV-B-induced NO generation and subsequent stomatal closure were inhibited by NO scavenger and NR inhibitors in guard cells. At the same time, exogenous NO donor reversed UV-B-induced stomatal closure in these plants [123]. Based on this observation, ET has been implicated as a signal acting upstream NO during UV-B-induced stomatal closure.

Various signalling pathways can be found in various plant organs and cell types upon presence or absence of light. It is well known that dark stimulates ET production and stomatal closure which is mediated by NO [124,125]. A positive correlation between the effects of ET and NO on stomatal movement has been observed in the dark. Both ethephon and ACC reduced NO levels in guard cells of *Vicia faba*, thus promoting stomatal opening in darkness [126]. In addition, ACC and ethephon suppressed the SNP-induced stomatal closure and NO levels in *Vicia* guard cells in the light [127]. In contrast, dose-dependent stomatal closure has been found after ET treatment under light condition, which was mediated by NR-dependent NO accumulation in *Vicia faba* guard cells [128]. It can be concluded that depending on the light intensity ET induces stomatal opening or closure through influencing the level of NO in guard cells.

### 3.2. NO–ET Crosstalk during Temperature Stress Responses

Both low temperature (cold and freezing) stress and heat stress can seriously affect plant growth and development. Plants have evolved sophisticated mechanisms involving altered molecular, biochemical and physiological processes to tolerate temperature stresses, in which ET and NO are key components, but their interaction remains to be elucidated [129,130]. 

The NO donor SNP has been shown to induce the expression of *MfSAMS1* and resulted in an elevated SAM level, PA concentration and PA oxidation under cold stress (5 °C) in *Medicago sativa* [131]. However, altered ET emission has been found in parallel with the enhanced tolerance to cold stress. This report indicates that SAMS plays an important role in plant tolerance upon cold stress via up-regulating PA oxidation and improving hydrogen peroxide (H_2_O_2_)-induced antioxidant protection [131]. Although it was reported that NO increases cold tolerance, the role of SAM, (a common precursor of PA and ET) in cold stress is in not well understood.

In line with the above described antagonistic relationship between ET-NO during fruit ripening (see Section 2.3), accumulating evidence also indicates a close interaction between these two gasotransmitters in fruit cold stress resistance [81]. For example, treatments with different dosages of NO fumigation (5, 10, 20 and 40 µL L^−1^) significantly decreased ET production during fruit ripening after 2 and 4 weeks of cold storage, reduced chilling injury and delayed fruit colour development, as well as softening and ripening in cold-stored mango fruits [132]. Similar changes have been described in Yali pears (*Pyrus bretschneideri*), since ET production and soluble sugar content decreased as the effect of NO fumigation after 60 days at 0 °C, and the softening and ripening of fruits were simultaneously delayed [133]. Others have also found that ET and antioxidant enzyme (superoxide dismutase, SOD; CAT; peroxidase, POD) activity was reduced after NO fumigation in the 4-week cold-stored peach fruits [134] whereas sucrose phosphate synthase activity increased resulting in a higher sucrose content of peach fruit under cold condition [135]. Therefore, an antagonism between NO and ET seems to play a critical role during fruit cold storage, delaying many aspects of fruit senescence and impacting fruit quality. 

Heat stress is also a serious agricultural problem in many areas of the world, limiting productivity due to negative impacts on plant photosynthesis, respiration, water relations, and membrane stability. Moreover, heat stress affects membrane fluidity, metabolism, and cytoskeleton rearrangement, as well as it results in the accumulation of unfolded proteins, and impacts the production of ROS, NO and multiple phytohormones, including ET [136]. Despite the several lines of evidence implicating NO in heat stress resistance (reviewed by [130]), the link between NO and ET under heat stress remains poorly explored [130]. Nevertheless, it is currently known that increased NO production and decreased ET emission can be promoted after 2-h-long heat stress (37 °C) in alfalfa (*Medicago sativa*) plantlets [131]. 

### 3.3. NO–ET Interplay during Drought Stress Responses

Drought stress induces changes in the osmotic homeostasis and causes loss of turgidity in plant cells. At the organ level, rapid closure of stomata can reduce the additional water loss, but it decreases intracellular CO_2_ concentration and causes a decline in the photosynthetic rate, thus reducing the growth of drought-stressed plants. At the cellular level, synthesis of antioxidants, osmoprotectants, dehydrins, and late-embryogenesis abundant proteins serve as a tolerance mechanism regulated by phytohormones [137]. Although NO is a central player in drought stress tolerance by improving the antioxidant defence system and osmoprotectants [138], its interaction with ET has not gained much attention. It has been shown that ET and NO emissions were drastically reduced after 4-days-long drought stress in *Arabidopsis* [139]. Decreased NR activity was also detected under drought stress. At the same time, the mechanisms behind ET action during drought remains ambiguous as it can act either as a positive or negative regulator, depending on the tissue and conditions or the treatment duration. Significant expression of pathogenesis-related gene *TaBWPR-1.2* was observed after the application of ACC or SNP in the first and third days of drought stress in wheat (*Triticum aestivum*), and both molecules proved to play a role in aerenchyma formation in the seminal root cortex [140]. This observation underlines the involvement of ET and NO and their interaction in defence responses and cell death under abiotic stress. Nevertheless, the time-dependent NO–ET interaction can be crucial for developing tolerance against drought stress. Transient NO and ROS production and down-regulated *ETR1* expression were observed after treatment with non-protein amino acid β-aminobutyric acid, which improved the drought tolerance of potato (*Solanum tuberosum*) [140]. Furthermore, the role of other phytohormones in this interaction under drought stress has to be also considered. High concentration of ABA can limit ET production and increase NO in stomata [141,142]. It can be concluded that further studies are required to evaluate the time-dependent interaction between the two gasotransmitters under drought stress in different plant cells and organs.

### 3.4. NO–ET Interaction during Hypoxia

NO, ET, and Hbs interaction during hypoxia has also been described via mechanisms that have only recently started to be elucidated. The balance between nitrate and NO levels in plant tissues is regulated by the activities of NR and Hbs in the Hb/NO cycle, which can maintain ATP generation under hypoxia [143]. The generation of NO is intensified by low oxygen availability and the hypoxia-triggered NO activates ET biosynthesis, possibly through the regulation of ACS and ACO. The increased ET levels result in hyponasty via well-characterized mechanisms [144].

Under conditions when oxygen availability is limited, wheat plants were shown to produce NO through NR-dependent mechanism and this hypoxia-induced NO proved to be necessary for aerenchyma formation [145]. NO produced by hypoxic roots induced ACS and ACO genes and consequently increased ET levels. Furthermore, hypoxia-induced NO triggered events related to cell death, such as ROS production, lipid peroxidation, protein nitration, cellulase induction, DNA fragmentation. These pharmacological data evidenced the involvement of hypoxia-induced NO in aerenchyma formation in wheat roots [145]. Altogether, these data illustrate the positive effect of NO both on ET levels and ET-regulated responses (hyponasty, aerenchyma formation) during low oxygen availability.

### 3.5. NO–ET Interaction during Salt Stress Responses

Salt stress is one of the most harmful environmental stresses, particularly in arid and semiarid regions, which can disrupt cellular structures and impair physiological functions of plants leading to growth disturbance, reduced fertility, premature senescence, and yield loss or programmed cell death by inducing osmotic, ionic and nitro-oxidative stress [137,138,146]. Both NO and ET has been shown to act as a signalling molecule in this process, improving salt stress tolerance of plants through activating defence responses in various ways (reviewed by [147]).

The ET-NO interaction under salt stress has been investigated first in *Arabidopsis* callus [148]. NO treatment of salt-stress exposed callus reduced electrolyte leakage (EL) near to the control levels, but SNP had a weaker protective effect in *etr1-3* compared to WT callus. Another NO donor, GSNO was also able to reduce EL in WT callus under salt stress (100 mM NaCl). Plasma membrane H^+^-ATPase activity was increased by ACC and SNP and the Na^+^ content sharply reduced while K^+^ content slightly increased leading to a lower Na^+^ to K^+^ ratio in WT callus but not in *etr1-3* callus under salt stress. Rapid NO accumulation and ET emission were induced by salt stress in callus culture and NO production greatly stimulated ET production in WT callus cooperating in enhancing salt stress tolerance [148]. The concentration- and time-dependent effect of salt stress and the signalling role of ET and NO in this process were later investigated in tomato cell suspension culture [149,150], where dose-dependent effects of NaCl on NO production was observed. Treatment with ACC (10 µM) decreased NO generation upon both NaCl treatments and elevated EL under salt stress [149]. ACC increased both ROS and NO production already after 1 h and accelerated cell death in case of lethal salt stress. Furthermore, 2-(4-carboxyphenyl)-4,4,5,5-tetramethylimidazoline-1-oxyl-3-oxide (cPTIO) slightly and aminoethoxyvinylglycine (AVG) significantly reduced cell death, which was in accordance with the decrease in ROS production of cells exposed to high salinity [150].

However, the interaction between NO and ET can be dependent not only on the duration and intensity of salt stress [151] but also on the various plant cell types and organs which are exposed to salinity [152]. NO and ET productions were significantly induced by 100 and 250 mM NaCl in the roots of tomato WT and ET-receptor mutant *Never ripe* (*Nr*) plants. However, levels of both gasotransmitters markedly elevated just after 1 h then decreased in case of the lethal (250 mM) salt treatment. Stronger nitro-oxidative stress was observed in *Nr* roots under moderate (100 mM) salt stress, which led to the induction of programmed death of tissues, characterized by the DNA and protein degradation, by the decrease in K^+^/Na^+^ ratio and loss of cell viability [153]. 

NO and ET regulate not only cell death but other developmental processes under salt stress. Both SNP and ACC improved seed germination of *Arabidopsis* under salinity stress (150 mM NaCl for 2, 3 and 4 d) [154]. Also, it was observed that ACC increased NO production but decreased H_2_O_2_ content after NaCl treatment. Moreover, expression of the *ACS2* gene was induced by NO under 4-days-long salinity stress, suggesting that each substance influences the production of the other [155]. It was revealed that SNP alone or together with NaCl enhanced more effectively the expression of *EIN3* transcripts after 6 h, compared with *ACO4* and *ACS2* in *Arabidopsis* [154]. Moreover, the expression level of *ERF1* increased eightfold after 6 h of 200 mM NaCl treatment, which coincided with the EIN3 protein level in this plant. Authors concluded that SNP promoted seed germination and seedlings growth of salt-exposed *Arabidopsis* which may depend on transcription factor EIN3 protein [154]. The inhibitory effects of salt stress on plant growth and development evolved by high ET levels can be modulated by exogenous application of NO donors in leaves. In the leaves of wheat, the application of SNP under salt stress (100 mM NaCl) alleviated the effects of salt stress by decreasing glucose sensitivity and by reducing the stress ET to optimal level, which had beneficial effects on stomatal closure, photosynthetic activity (maximum quantum yield of PSII, net photosynthetic assimilation, Rubisco activity), proline synthesis, NR activity and antioxidant metabolism (SOD, CAT, APX, and glutathione reductase, GR) [155]. Despite the numerous physiological data about ET-NO interaction upon salt stress, the role of NO-mediated PTMs (tyrosine nitration, S-nitrosation) in plant responses to high and moderate salinity requires further research.

### 3.6. NO–ET Interplay during Plant Responses to Nutrient Deficiencies

Insufficient supply of essential nutrients like iron (Fe), phosphorus (P) and magnesium (Mg) has a wide negative impact both on plant growth and yield. Since the topic has agricultural relevance, the signal processes regulating plant responses to nutrient deficiencies are intensely studied. 

Followed by the observation that ET is involved in up-regulating Fe acquisition genes (e.g., *AtFIT, AtFRO2, AtIRT*, [156]), it was demonstrated that these genes were also responsive to NO treatment [157] suggesting an interplay between these gasotransmitters in Fe deficiency response of *Arabidopsis*. The signal interactions between NO and ET were further clarified when NO was shown to promote the expression of genes involved in ethylene synthesis in *Arabidopsis* and cucumber (e.g., *AtSAM1, AtACS6, AtACO1, CsACS2, CsACO2*) and ethylene increases NO level in the roots [158]. The authors argued that in Fe-deficient Strategy I plants, NO and ET synergistically induce the expression of Fe-acquisition genes, influence ferric reductase activity in roots, and they regulate each other’s levels in a mutually positive way [157]. Recently, this model was supplemented by the regulatory role of GSNO and GSNOR in Fe-deficient roots. ET (ACC) was found to increase GSNOR activity and expression leading to low GSNO level which, through unblocking ET synthesis, can maintain ET production in Fe-deficient roots [159].

In the case of P deficiency, rice roots showed rapid NO generation, which was followed by a slower ET emission [160]. Moreover, modifying NO levels (NO donor and scavenger treatments) resulted in altered ET production, while the modification of ET levels did not affect NO production of rice roots suggesting that NO acts upstream of ET in signalling of P deficiency response. Both gasotransmitters increased soluble P content in P-deficient rice roots and pharmacological experiments evidenced that ET acts downstream of NO in this signalling [160]. 

Root hair development was stimulated by Mg deficiency in *Arabidopsis,* and this process was accompanied by increased NO and ET generation [161]. Ethylene triggered NO synthesis by increasing the activities of both NR and NOS-like enzyme; however, we have to mention that the existence of the mammalian-like NOS activity in higher plants is highly questionable [17]. Also, NO increased ET synthesis through activating ACO and ACS enzymes. Furthermore, pharmacological treatments proved that inhibiting either the effect of NO or ET prevented the stimulation of root hair morphogenesis in Mg-deficient *Arabidopsis* [161]. These reports collectively demonstrate that both NO and ET is involved in plant responses to nutrient deficiencies and these gaseous molecules act synergistically and mutually influence each other’s levels.

### 3.7. NO–ET Interaction during Heavy Metal Stress Responses

Heavy metal (HM) contamination has become a worldwide environmental concern with a damaging impact on agriculture. At the cellular level, the elevated quantity of HMs causes oxidative stress, which induces the disruption of the redox homeostasis of cells and at high level leads to tissue damage and morpho-physiological changes in plants. The major signalling networks working in metal stresses are among others calcium, MAPK and hormone signalling [162]. Thus, NO and ET also influence plant responses to metal stress, like arsenite (AsIII), arsenate (AsV), cadmium (Cd), copper (Cu), nickel (Ni), lead (Pb), or zinc (Zn) (reviewed by [138] and [163]). 

Although the role of both NO and ET have been widely studied under HM stress, NO–ET crosstalk has been analysed only upon excess Cd. The effect of Cd on the metabolism of ROS and NO in parallel with changes in JA, SA, and ET was studied in pea (*Pisum sativum*) by [164,165]. In these works, significant JA, SA, and ET production, high ROS levels and lower NO levels were registered upon Cd stress, which was consistent with Cd-induced senescence. Based on the suggested model, authors emphasize that ET and NO have antagonistic effects in the roots and leaves in the presence of Cd, since Cd-triggered nutritional imbalance causes NO level decrease and consequent changes in protein nitrosation which may positively affect ET synthesis [165]. Interestingly, others reported that short term treatments with Cd elevated the expression of genes encoding proteins involved both in ET (ACS) and NO synthesis (NR) in young soybean (*Glycine max*) seedlings [166]. Our knowledge about HM-induced NO and ET production by plant tissues is limited, as well as their interactions at the biochemical and molecular level and also their roles in plant responses to excess metals are hardly understood. Therefore, researchers need to pay more attention to examine NO–ET interplay in heavy metal-exposed plants.

## 4. Conclusions and Future Perspectives

Both NO and ET are gaseous signals sharing common regulatory roles but showing different characteristics. The synthesis, perception and signalling of ET are well-characterized, whereas the plant enzymatic synthesis of NO remains obscure and our knowledge on its perception and signalling is largely incomplete.

The available literature data indicate that NO and ET are synthetized by plants in various developmental stages (e.g., seeds, fruits) and as a response to environmental factors (e.g., heat, heavy metals). Furthermore, NO and ET mutually influence each other’s levels as revealed mostly by pharmacological experiments (NO donors and scavengers, ET precursors and biosynthesis inhibitors). The nature of NO–ET crosstalk can be synergistic and also antagonistic, and there is experimental evidence for both types of interactions. Interestingly, most of the growth and developmental processes (e.g., fruit ripening, de-etiolation) are regulated by NO–ET antagonism, while in abiotic stress responses the picture is more complex. Beyond antagonistic interplays, several stress responses (e.g., UV-B-induced stomatal closure, P deficiency-induced P remobilization, lethal salt stress-induced cell death) are modulated by NO–ET synergism.

Reviewing the literature shows that the NO–ET link has been actively studied in some processes (e.g., fruit ripening, responses to salt stress and nutrient deficiencies), while other processes (e.g., floral transition, root and shoot development, heat stress responses) are far more underestimated in point of view NO and ET research. Most research applies pharmacological approaches in order to reveal the effects of NO and ET on each other and on the physiological process studied; however, these experiments should be completed with investigating mutant or transgenic plants with altered NO and/or ET metabolism.

Moreover, further research into the organ-level and temporal regulation of NO–ET and gene expression is necessary in order to understand the mechanisms of their regulation in developmental and stress-regulated processes. Further research on NO-dependent posttranslational modifications (primarily S-nitrosation) of proteins involved in ET synthesis and signalling would give new insights into their interactions.

Understanding the mechanisms of NO–ET interactions at the molecular and physiological levels is relevant because this knowledge can contribute to increment yield and intensify stress tolerance of crop plants in an ever-changing environment.

## References

[1] Lancaster J.R. (2015). Nitric oxide: A brief overview of chemical and physical properties relevant to therapeutic applications. Future Sci. OA.

[2] Wellburn A.R., Majernik O., Wellburn A.M. (1972). Effects of SO_2_ and NO_2_ polluted air upon the ultra structure of the chloroplast. Environ. Pollut..

[3] Benett J.H., Hill A.C. (1973). Inhibition of apparent photosynthesis by air pollutants. J. Environ. Qual..

[4] Taylor O.C., Dugger M. (1974). Air pollutant effects influenced by plant environmental interactions. Air Pollution Effects on Plant Growth.

[5] Zeewart A.J. (1976). Some effects of fumigating plants for short periods with NO_2_. Environ. Pollut..

[6] Bakshi A., Shemansky J.M., Chang C., Binder B.M. (2015). History of research on the plant hormone ethylene. J. Plant Growth Regul..

[7] Burg S.P., Burg E.A. (1966). The interaction between auxin and ethylene and its role in plant growth. Proc. Natl. Acad. Sci. USA.

[8] Ku H.S., Suge H., Rappaport L., Pratt H.K. (1970). Stimulation of rice coleoptile growth by ethylene. Planta.

[9] Apelbaum A., Stanley P., Burg S.P. (1972). Effect of ethylene on cell division and deoxyribonucleic acid synthesis in *Pisum sativum*. Plant Physiol..

[10] Burg S.P. (1973). Ethylene in plant growth. Proc. Natl. Acad. Sci. USA.

[11] Sanz-Luque E., Ocaña-Calahorro F., Llamas A., Galvan A., Fernandez E. (2013). Nitric oxide controls nitrate and ammonium assimilation in *Chlamydomonas reinhardtii*. J. Exp. Bot..

[12] Rockel P., Strube F., Rockel A., Wildt J., Kaiser W.M. (2002). Regulation of nitric oxide (NO) production by plant nitrate reductase In Vivo and In Vitro. J. Exp. Bot..

[13] Chamizo-Ampudia A., Sanz-Luque E., Llamas Á., Ocaña-Calahorro F., Mariscal V., Carreras A., Barroso J.B., Galván A., Fernández E. (2016). A dual system formed by the ARC and NR molybdoenzymes mediates nitrite-dependent NO production in *Chlamydomonas*. Plantcell Environ..

[14] Stöhr C., Strube F., Marx G., Ullrich W.R., Rockel P. (2001). A plasma membrane-bound enzyme of tobacco roots catalyses the formation of nitric oxide from nitrite. Planta.

[15] Bethke P.C., Badger M.R., Jones R.L. (2004). Apoplastic synthesis of nitric oxide by plant tissues. Plant Cell.

[16] Corpas F.J., Palma J.M., Del Río L.A., Barroso J.B. (2009). Evidence supporting the existence of L-arginine-dependent nitric oxide synthase activity in plants. New Phytol..

[17] Hancock J.T., Neill S.J. (2019). Nitric Oxide: Its generation and interactions with other reactive signaling compounds. Plants.

[18] Corpas F.J., Barroso J.B. (2017). Nitric oxide synthase-like activity in higher plants. Nitric Oxide Biol. Chem..

[19] Foresi N., Correa-Aragunde N., Parisi G., Caló G., Salerno G., Lamattina L. (2010). Characterization of a nitric oxide synthase from the plant kingdom: NO generation from the green alga *Ostreococcus tauri* is light irradiance and growth phase dependent. Plant Cell.

[20] Correa-Aragunde N., Foresi N., Del Castello F., Lamattina L.A. (2018). singular nitric oxide synthase with a globin domain found in *Synechococcus* PCC 7335 mobilizes N from arginine to nitrate. Sci. Rep..

[21] Wimalasekera R., Villar C., Begum T., Scherer G.F.E. (2011). Copper Amine Oxidase1 (CuAO1) of *Arabidopsis thaliana* contributes to abscisic acid-and polyamine-induced nitric oxide biosynthesis and abscisic acid signal transduction. Mol. Plant.

[22] Groß F., Rudolf E.-E., Thiele B., Durner J., Astier J. (2017). Copper amine oxidase 8 regulates arginine-dependent nitric oxide production in *Arabidopsis thaliana*. J. Exp. Bot..

[23] Panagiotis M.N., Aziz A., Kalliopi R.A.A., Hernâni Gerós M., Chaves M., Delrot S. (2012). Polyamines and Grape Berry Development. The Biochemistry of the Grape Berry.

[24] Wang K.L., Li H., Ecker J.R. (2002). Ethylene biosynthesis and signaling networks. Plant Cell.

[25] Beckman J.S., Beckman T.W., Chen J., Marshall P.A., Freeman B.A. (1990). Apparent hydroxyl radical production by peroxynitrite: Implication for endothelial injury from nitric oxide and superoxide. Proc. Natl. Acad. Sci. USA.

[26] Perazzolli M., Dominici P., Romero-Puertas M.C., Zago E., Zeier J., Sonoda M., Delledonne M. (2004). *Arabidopsis* nonsymbiotic hemoglobin AHb1 modulates nitric oxide bioactivity. Plant Cell.

[27] Hebelstrup K.H., Hunt P., Dennis E., Jensen S.B., Jensen E.Ø. (2006). Hemoglobin is essential for normal growth of *Arabidopsis* organs. Physiol. Plant.

[28] Chamizo-Ampudia A., Sanz-Luque E., Llamas A., Galvan A., Fernandez E. (2017). Nitrate reductase regulates plant nitric oxide homeostasis. Trends Plant Sci..

[29] Hogg N. (2000). Biological chemistry and clinical potential of S-nitrosothiols. Free Radic. Biol. Med..

[30] Foster M.W., McMahon T.J., Stamler J.S. (2003). S-nitrosylation in health and disease. Trends Mol. Med..

[31] Stamler J.S., Lamas S., Fang F.C. (2001). Nitrosylation: The prototypic redox-based signaling mechanism. Cell.

[32] Janhová J., Luhová L., Petřivalsky M. (2019). S-nitrosoglutathione reductase-The master regulator of protein S-nitrosation in plant NO signaling. Plants.

[33] Lindermayr C. (2018). Crosstalk between reactive oxygen species and nitric oxide in plants: Key role of S-nitrosoglutathione reductase. Free Radic. Biol. Med..

[34] Park J., Lee Y., Martinoia E., Geisler M. (2017). Plant hormone transporters: What we know and what we would like to know. BMC Biol..

[35] Umbreen S., Lubega J., Cui B., Pan Q., Jiang J., Loake G.J. (2018). Specificity in nitric oxide signaling. J. Exp. Bot..

[36] Lamotte O., Bertoldo J.B., Besson-Bard A., Rosnoblet C., Aimé S., Hichami S., Terenzi H., Wendehenne D. (2015). Protein S-nitrosylation: Specificity and identification strategies in plants. Front. Chem..

[37] Hu J., Huang X., Chen L., Sun X., Lu C., Zhang L., Wang Y., Zuo J. (2015). Site-specific nitrosoproteomic identification of endogenously S-nitrosylated proteins in *Arabidopsis*. Plant Physiol..

[38] Souza J.M., Peluffo G., Radi R. (2008). Protein tyrosine nitration—Functional alteration or just a biomarker?. Free Radic. Biol. Med..

[39] Kolbert Z.S., Feigl G., Bordé Á., Molnár Á., Erdei L. (2017). Protein tyrosine nitration in plants: Present knowledge, computational prediction and future perspectives. Plant Physiol. Biochem..

[40] Lozano-Juste J., Colom-Moreno R., León J. (2011). In vivo protein tyrosine nitration in *Arabidopsis thaliana*. J. Exp. Bot..

[41] Russwurm M., Koesling D. (2004). NO activation of guanylyl cyclase. EMBO J..

[42] Gibbs D.J., Md Isa N., Movahedi M., Lozano-Juste J., Mendiondo G.M., Berckhan S., Marín-de la Rosa N., Conde J.V., Correia C.S., Pearce S.P. (2014). Nitric oxide sensing in plants is mediated by proteolytic control of group VII ERF transcription factors. Mol. Cell.

[43] Chang C. (2016). Q and A: How do plants respond to ethylene and what is its importance?. BMC Biol..

[44] Bogatek R., Gniazdowska A. (2018). Ethylene in seed development, dormancy and germination. Annu. Plant Rev. Online.

[45] Bewley J.D., Black M. (1994). Seeds: Physiology of Development and GermiNatlion.

[46] Tuan P.A., Sun M., Nguyen T.-N., Park S., Ayele B.T. (2019). Molecular mechanisms of seed germination. Sprouted Grains.

[47] Kucera B., Cohn M.A., Leubner-Metzger G. (2005). Plant hormone interactions during seed dormancy release and germination. Seed Sci. Res..

[48] Matilla A.J., Matilla-Vázquez M.A. (2008). Involvement of ethylene in seed physiology. Plant Sci..

[49] Beligni M.V., Lamattina L. (2000). Nitric oxide stimulates seed germination and de-etiolation, and inhibits hypocotyl elongation, three light-inducible responses in plants. Planta.

[50] Sarath G., Bethke P.C., Jones R., Baird L.M., Hou G., Mitchell R.B. (2006). Nitric oxide accelerates seed germination in warm-season grasses. Planta.

[51] Bethke P.C., Libourel I.G.L., Aoyama N., Chung Y.Y., Still D.W., Jones R.L. (2007). The *Arabidopsis* aleurone layer responds to nitric oxide, gibberellin, and abscisic acid and is sufficient and necessary for seed dormancy. Plant Physiol..

[52] Arc E., Sechet J., Corbineau F., Rajjou L., Marion-Poll A. (2013). ABA crosstalk with ethylene and nitric oxide in seed dormancy and germination. Front. Plant Sci..

[53] Bethke P.C., Libourel I.G.L., Jones R.L. (2006). Nitric oxide reduces seed dormancy in *Arabidopsis*. J. Exp. Bot..

[54] Gniazdowska A., Dobrzyńska U., Babańczyk T., Bogatek R. (2007). Breaking the apple embryo dormancy by nitric oxide involves the stimulation of ethylene production. Planta.

[55] Gniazdowska A., Krasuska U., Bogatek R. (2010). Dormancy removal in apple embryos by nitric oxide or cyanide involves modifications in ethylene biosynthetic pathway. Planta.

[56] Kępczyński J., Cembrowska-Lech D., Sznigir P. (2017). Interplay between nitric oxide, ethylene, and gibberellic acid regulating the release of *Amaranthus retroflexus* seed dormancy. Acta Physiol. Plant.

[57] Iqbal N., Khan N.A., Ferrante A., Trivellini A., Francini A., Khan M.I.R. (2017). Ethylene role in plant growth, development and senescence: Interaction with other phytohormones. Front. Plant Sci..

[58] Freschi L. (2013). Nitric oxide and phytohormone interactions: Current status and perspectives. Front. Plant Sci..

[59] Melo N.K., Bianchetti R.E., Lira B.S., Oliveira P.M., Zuccarelli R., Dias D.L., Demarco D., Peres L.E., Rossi M., Freschi L. (2016). Nitric oxide, ethylene, and auxin cross talk mediates greening and plastid development in deetiolating tomato seedlings. Plant Physiol..

[60] Castillo M.-C., Coego A., Costa-Broseta Á., León J. (2018). Nitric oxide responses in *Arabidopsis* hypocotyls are mediated by diverse phytohormone pathways. J. Exp. Bot..

[61] Ivanchenko M.G., Muday G.K., Dubrovsky G. (2008). Ethylene–auxin interactions regulate lateral root initiation and emergence in *Arabidopsis thaliana*. Plant J..

[62] Negi S., Ivanchenko M.G., Muday G.K. (2008). Ethylene regulates lateral root formation and auxin transport in *Arabidopsis thaliana*. Plant J..

[63] Correa-Aragunde N., Graziano M. (2004). Lamattina Nitric oxide plays a central role in determining lateral root development in tomato. Planta.

[64] Singh N., Bhatla S.C. (2018). Nitric oxide regulates lateral root formation through modulation of ACC oxidase activity in sunflower seedlings under salt stress. Plant Signal. Behav..

[65] Feigl G., Horváth E., Molnár Á., Oláh D., Poór P., Kolbert Z.S. (2019). Ethylene-nitric oxide interplay during selenium-induced lateral root emergence in *Arabidopsis*. J. Plant Growth Regul..

[66] Pagnussat G.C., Simontacchi M., Puntarulo S., Lamattina L. (2002). Nitric oxide is required for root organogenesis. Plant Physiol..

[67] Robbins J.A., Kays S.J., Dirr M.A. (1983). Enhanced rooting of wounded mung bean cuttings by wounding and ethephon. J. Am. Soc. Hortic. Sci..

[68] Pan R., Wang J., Tian X. (2002). Influence of ethylene on adventitious root formation in mung bean hypocotyl cuttings. Plant. Growth Regul..

[69] Jin X., Liao W.B., Yu J.H., Ren P.J., Dawuda M.M., Wang M., Niu L.J., Li X.P., Xu X.T. (2017). Nitric oxide is involved in ethylene-induced adventitious rooting in marigold (*Tagetes erecta* L.). Can. J. Plant. Sci..

[70] Novikova G.V., Mur L.A.J., Nosov A.V., Fomenkov A.A., Mironov K.S., Mamaeva A.S., Shilov E.S., Rakitin V.Y., Hall M.A. (2017). Nitric oxide has a concentration-dependent effect on the cell cycle acting via EIN2 in *Arabidopsis thaliana* cultured cells. Front. Physiol..

[71] Achard P., Baghour M., Chapple A., Hedden P., Van Der Straeten D., Genschik P., Moritz T., Harberd N.P. (2007). The plant stress hormone ethylene controls floral transition via DELLA-dependent regulation of floral meristem-identity genes. Proc. Natl. Acad. Sci. USA.

[72] He Y.K., Tang R.H., Hao Y., Stevens R.D., Cook C.W., Am S.M., Jing L.F., Yang Z.G., Chen L.G., Guo F.Q. (2004). Nitric oxide represses the *Arabidopsis* floral transition. Science.

[73] Seligman K., Saviani E.E., Oliveira H.C., Pinto-Maglio C.A.F., Salgado I. (2008). Floral transition and nitric oxide emission during flower development in *Arabidopsis thaliana* is affected in nitrate reductase-deficient plants. Plant. Cell Physiol..

[74] Kwon E., Feechan A., Yun B.-W., Hwang B.-H., Pallas J.A., Kang J.-G., Loake G.J. (2012). *AtGSNOR1* function is required for multiple developmental programs in *Arabidopsis*. Planta.

[75] Prado A.M., Colaco R., Moreno N., Silva A.C., Feijo J.A. (2008). Targeting of pollen tubes to ovules is dependent on nitric oxide (NO) signaling. Mol. Plant..

[76] Bodanapu R., Gupta S.K., Basha P.O., Sakthivel K., Sadhana Sreelakshmi Y., Sharma R. (2016). Nitric oxide overproduction in tomato *shr* mutant shifts metabolic profiles and suppresses fruit growth and ripening. Front. Plant Sci..

[77] Jia H.L., Yang J., Liesche J., Liu X., Hu Y.F., Si W.T., Guo J.K., Li J.S. (2018). Ethylene promotes pollen tube growth by affecting actin filament organization via the cGMP-dependent pathway in *Arabidopsis thaliana*. Protoplasma.

[78] Carbonell-Bejerano P., Urbez C., Granell A., Carbonell J., Perez-Amador M.A. (2011). Ethylene is involved in pistil fate by modulating the onset of ovule senescence and the GA-mediated fruit set in *Arabidopsis*. BMC Plant. Biol..

[79] Shinozaki Y., Hao S., Kojima M., Sakakibara H., Ozeki-Iida Y., Zheng Y., Fei Z., Zhong S., Giovannoni J.J., Rose J.K. (2015). Ethylene suppresses tomato (*Solanum lycopersicum*) fruit set through modification of gibberellin metabolism. Plant. J..

[80] Leshem Y.Y., Haramaty E. (1996). The characterization and contrasting effects of the nitric oxide free radical in vegetative stress and senescence of *Pisum sativum* Linn. Foliage. J. Plant. Physiol..

[81] Leshem Y.Y., Pinchasov Y. (2000). Non-invasive photoacoustic spectroscopic determination of relative endogenous nitric oxide and ethylene content stoichiometry during the ripening of strawberries *Fragaria anannasa* (Duch.) and avocados *Persea americana* (Mill.). J. Exp. Bot..

[82] Mukherjee S. (2019). Recent advancements in the mechanism of nitric oxide signaling associated with hydrogen sulfide and melatonin crosstalk during ethylene-induced fruit ripening in plants. Nitric Oxide.

[83] Corpas F.J., Freschi L., Rodriguez-Ruiz M., Mioto P.T., Gonzalez-Gordo S., Palma J.M. (2018). Nitro-oxidative metabolism during fruit ripening. J. Exp. Bot..

[84] Manjunatha G., Lokesh V., Neelwarne B. (2010). Nitric oxide in fruit ripening: Trends and opportunities. Biotechnol. Adv..

[85] Cruz A.B., Bianchetti R.E., Alves F.R.R., Purgatto E., Peres L.E.P., Rossi M., Freschi L. (2018). Light, ethylene and auxin signaling interaction regulates carotenoid biosynthesis during tomato fruit ripening. Front. Plant Sci..

[86] Kader A.A., Kader A.A. (2002). Postharvest biology and technology: An overview. Postharvest Technology of Horticultural Crops.

[87] Eum H.L., Kim H.B., Choi S.B., Lee S.K. (2009). Regulation of ethylene biosynthesis by nitric oxide in tomato *(Solanum lycopersicum* L.) fruit harvested at different ripening stages. Eur. Food Res. Technol..

[88] Rodríguez-Ruiz M., Mateos R.M., Codesido V., Corpas F.J., Palma J.M. (2017). Characterization of the galactono-1, 4-lactone dehydrogenase from pepper fruits and its modulation in the ascorbate biosynthesis. Role of nitric oxide. Redox Biol..

[89] Rodríguez-Ruiz M., Zuccarelli R., Palma J.M., Corpas F.J., Freschi L., Gupta D.K., Palma J.M., Corpas F.J. (2019). Biotechnological Application of Nitric Oxide and Hydrogen Peroxide in Plants. Nitric Oxide and Hydrogen Peroxide Signaling in Higher Plants.

[90] Parra-Lobato M.C., Gómez-Jiménez M.C. (2011). Polyamine-induced modulation of genes involved in ethylene biosynthesis and signaling pathways and nitric oxide production during olive mature fruit abscission. J. Exp. Bot..

[91] Chaki M., Álvarez de Morales P., Ruiz C., Begara-Morales J.C., Barroso J.B., Corpas F.J., Palma J.M. (2015). Ripening of pepper (*Capsicum annuum*) fruit is characterized by an enhancement of protein tyrosine nitration. Ann. Bot..

[92] Rodriguez-Ruiz M., Mioto P., Palma J.M., Corpas F.J. (2017). S-nitrosoglutathione reductase (GSNOR) activity is down-regulated during pepper (*Capsicum annuum* L.) fruit ripening. Nitric Oxide.

[93] Rudell D.R., Mattheis J.P. (2006). Nitric oxide and nitrite treatments reduce ethylene evolution from apple fruit disks. HortScience.

[94] Cheng G., Yang E., Lu W., Jia Y., Jinag Y., Duan X. (2009). Effect of nitric oxide on ethylene synthesis and softening of banana fruit slice during ripening. J. Agric. Food Chem..

[95] Guo Q., Wu B., Chen W., Zhang Y., Wang J., Li X. (2014). Effects of nitric oxide treatment on the cell wall softening related enzymes and several hormones of papaya fruit during storage. Food Sci. Technol. Int..

[96] Zhu S.H., Liu M.C., Zhou J. (2006). Inhibition by nitric oxide of ethylene biosynthesis and lipoxygenase activity in peach fruit during storage. Postharvest Biol. Technol..

[97] Zaharah S.S., Singh Z. (2011). Mode of action of nitric oxide in inhibiting ethylene biosynthesis and fruit softening during ripening and cool storage of ‘Kensington Pride’ mango. Postharvest Biol. Technol..

[98] Lindermayr C., Saalbach G., Durner J. (2005). Proteomic identification of S-nitrosylated proteins in Arabidopsis. Plant. Physiol..

[99] Abat J.K., Mattoo A.K., Deswal R. (2008). S-nitrosylated proteins of a medicinal CAM plant *Kalanchoe pinnata*—ribulose-1, 5-bisphosphate carboxylase/oxygenase activity targeted for inhibition. FEBS J..

[100] Abat J.K., Deswal R. (2009). Differential modulation of S-nitrosoproteome of *Brassica juncea* by low temperature: Change in S-nitrosylation of Rubisco is responsible for the inactivation of its carboxylase activity. Proteomics.

[101] Lindermayr C., Saalbach G., Bahnweg G., Durner J. (2006). Differential inhibition of *Arabidopsis* methionine adenosyltransferases by protein S-nitrosylation. J. Biol. Chem..

[102] Correa-Aragunde N., Foresi N., Lamattina L. (2015). Nitric oxide is a ubiquitous signal for maintaining redox balance in plant cells: Regulation of ascorbate peroxidase as a case study. J. Exp. Bot..

[103] Corpas F.J., Gonzalez-Gordo S., Canas A., Palma J.M. (2019). Nitric oxide (NO) and hydrogen sulfide (H_2_S) in plants: Which is first?. J. Exp. Bot..

[104] Huo J., Huang D., Zhang J., Fang H., Wang B., Wang C., Liao W. (2018). Hydrogen sulfide: A gaseous molecule in postharvest freshness. Front. Plant Sci..

[105] Ma N., Ma C., Liu Y., Shahid M.O., Wang C., Gao J. (2018). Petal senescence: A hormone view. J. Exp. Bot..

[106] Badiyan D., Wills R.B.H., Bowyer M.C. (2004). Use of a nitric oxide donor compound to extend the vase life of cut flowers. HortScience.

[107] Guo F.Q., Crawford N.M. (2005). *Arabidopsis* nitric oxide synthase1 is targeted to mitochondria and protects against oxidative damage and dark-induced senescence. Plant. Cell.

[108] Mishina T.E., Lamb C., Zeier J. (2007). Expression of a nitric oxide degrading enzyme induces a senescence programme in *Arabidopsis*. Plant Cell Environ..

[109] Corpas F.J., Barroso J.B., Carreras A., Quiros M., Leon A.M., Romero-Puertas M.C., Esteban F.J., Valderrama R., Palma J.M., Sandalio L.M. (2004). Cellular and subcellular localization of endogenous nitric oxide in young and senescent pea plants. Plant. Physiol..

[110] Lv S.-F., Jia M.-Z., Zhang S.-S., Han S., Jiang J. (2019). The dependence of leaf senescence on the balance between 1-aminocyclopropane-1-carboxylate acid synthase 1 (ACS1)-catalyzed ACC generation and nitric oxide associated 1 (NOS1)-dependent NO accumulation in *Arabidopsis*. Plant. Biol..

[111] Leshem Y.Y., Wills R.B., Ku V.V.V. (1998). Evidence for the function of the free radical gas—Nitric oxide (NO•)—as an endogenous maturation and senescence regulating factor in higher plants. Plant. Physiol. Biochem..

[112] Miller J.D., Arteca R.N., Pell E.J. (1999). Senescence-associated gene expression during ozone-induced leaf senescence in *Arabidopsis*. Plant. Physiol..

[113] Niu Y.-H., Guo F.-Q. (2012). Nitric oxide regulates dark-induced leaf senescence through EIN2 in *Arabidopsis*. J. Integr. Plant. Biol..

[114] Alonso J.M., Hirayama T., Roman G., Nourizadeh S., Ecker J.R. (1999). EIN2, a bifunctional transducer of ethylene and stress responses in *Arabidopsis*. Science.

[115] Liao W.B., Zhang M.L., Yu J.H. (2013). Role of nitric oxide in delaying senescence of cut rose flowers and its interaction with ethylene. Sci. Hortic..

[116] De Wit M., Galvao V.C., Fankhauser C. (2016). Light-mediated hormonal regulation of plant growth and development. Annu. Rev. Plant. Biol..

[117] Takahashi S., Badger M.R. (2011). Photoprotection in plants: A., n.e.w.light on photosystem II damage. Trends Plant. Sci..

[118] Demarsy E., Goldschmidt-Clermont M., Ulm R. (2018). Coping with ‘dark sides of the sun’ through photoreceptor signaling. Trends Plant. Sci..

[119] Magalhaes J.R., Monte D.C., Durzan D. (2000). Nitric oxide and ethylene emission in *Arabidopsis thaliana*. Physiol. Mol. Biol. Plants.

[120] Mackerness A.H.S., John C.F., Jordan B., Thomas B. (2001). Early signaling components in ultraviolet-B responses: Distinct roles for different reactive oxygen species and nitric oxide. FEBS Lett..

[121] Vanhaelewyn L., Prinsen E., Van Der Straeten D., Vandenbussche F. (2016). Hormone-controlled UV-B responses in plants. J. Exp. Bot..

[122] Wang Y., Feng H., Qu Y., Cheng J., Zhao Z., Zhang M., Wang X., An L. (2006). The relationship between reactive oxygen species and nitric oxide in ultraviolet-B-induced ethylene production in leaves of maize seedlings. Environ. Exp. Bot..

[123] He J.M., Zhang Z., Wang R.B., Chen Y.P. (2011). UV-B-induced stomatal closure occurs via ethylene-dependent NO generation in *Vicia faba*. Funct. Plant Biol..

[124] She X.P., Son X.G., He J.M. (2004). Role and relationship of nitric oxide and hydrogen peroxide in light/dark-regulated stomatal movement in *Vicia faba*. Acta Bot. Sin.-Engl. Ed..

[125] Huang A.X., Wang Y.S., She X.P., Mu J., Zhao J.L. (2015). Copper amine oxidase-catalysed hydrogen peroxide involves production of nitric oxide in darkness-induced stomatal closure in broad bean. Funct. Plant Biol..

[126] Song X.G., She X.P., Wang J., Sun Y.C. (2011). Ethylene inhibits darkness-induced stomatal closure by scavenging nitric oxide in guard cells of *Vicia faba*. Funct. Plant Biol..

[127] She X., Song X. (2012). Ethylene inhibits abscisic acid-induced stomatal closure in *Vicia faba* via reducing nitric oxide levels in guard cells. N. Z. J. Bot..

[128] Liu J., Hou Z.H., Liu G.H., Hou L.X., Liu X. (2012). Hydrogen sulfide may function downstream of nitric oxide in ethylene-induced stomatal closure in *Vicia faba* L.. J. Integr. Agric..

[129] Majláth I., Szalai G., Soós V., Sebestyén E., Balázs E., Vanková R., Dobrev P.I., Tari I., Tandori J., Janda T. (2012). Effect of light on the gene expression and hormonal status of winter and spring wheat plants during cold hardening. Physiol. Plant.

[130] Parankusam S., Adimulam S.S., Bhatnagar-Mathur P., Sharma K.K. (2017). Nitric oxide (NO) in plant heat stress tolerance: Current knowledge and perspectives. Front. Plant Sci..

[131] Guo Z., Tan J., Zhuo C., Wang C., Xiang B., Wang Z. (2014). Abscisic acid, H_2_O_2_ and nitric oxide interactions mediated cold-induced S-adenosylmethionine synthetase in *Medicago sativa* subsp. falcata that confers cold tolerance through up-regulating polyamine oxidation. Plant. Biotechnol. J..

[132] Zaharah S.S., Singh Z. (2011). Postharvest nitric oxide fumigation alleviates chilling injury, delays fruit ripening and maintains quality in cold-stored ‘Kensington Pride’ mango. Postharvest Biol. Technol..

[133] Liu L.Q., Yu D., Guan J.F. (2011). Effects of nitric oxide on the quality and pectin metabolism of Yali pears during cold storage. Agric. Sci. China.

[134] Tareen M.J., Singh Z., Khan A.S., Abbasi N.A., Naveed M. (2017). Combined applications of Aminoethoxyvinylglycine with salicylic acid or nitric oxide reduce oxidative stress in peach during ripening and cold storage. J. Plant. Growth Regul..

[135] Han S., Cai H., An X., Huan C., Wu X., Jiang L., Mingliang Y., Ruijuan M., Yu Z. (2018). Effect of nitric oxide on sugar metabolism in peach fruit (cv. Xiahui 2018, 6) during cold storage. Postharvest Biol. Technol..

[136] Wahid A., Gelani S., Ashraf M., Foolad M.R. (2007). Heat tolerance in plants: An overview. Environ. Exp. Bot..

[137] Peleg Z., Blumwald E. (2011). Hormone balance and abiotic stress tolerance in crop plants. Curr. Opin. Plant. Biol..

[138] Zhu J.K. (2016). Abiotic stress signaling and responses in plants. Cell.

[139] Nabi R.B.S., Tayade R., Hussain A., Kulkarni K.P., Imran Q.M., Mun B.G., Yu B.W. (2019). Nitric oxide regulates plant responses to drought, salinity, and heavy metal stress. Environ. Exp. Bot..

[140] Haque M.E., Abe F., Mori M., Oyanagi A., Komatsu S., Kawaguchi K. (2014). Characterization of a wheat pathogenesis-related protein, TaBWPR-1.2, in seminal roots in response to waterlogging stress. J. Plant Physiol..

[141] Sós-Hegedűs A., Juhász Z., Poór P., Kondrák M., Antal F., Tari I., Mauch-Mani B., Bánfalvi Z. (2014). Soil drench treatment with ß-aminobutyric acid increases drought tolerance of potato. PLoS ONE.

[142] Wilkinson S., Davies W.J. (2010). Drought, ozone, ABA and ethylene: New insights from cell to plant to community. Plant Cell Environ..

[143] Igamberdiev A.U., Baron K., Manac’h-Little N., Stoimenova M., Hill R.D. (2005). The Haemoglobin/Nitric Oxide Cycle: Involvement in flooding stress and effects on hormone signalling. Ann. Bot..

[144] Hebelstrup K.H., van Zanten M., Mandon J., Voesenek L.A., Harren F.J., Cristescu S.M., Møller I.M., Mur L.A. (2012). Haemoglobin modulates NO emission and hyponasty under hypoxia-related stress in *Arabidopsis thaliana*. J. Exp. Bot..

[145] Wany A., Kumari A., Gupta K.J. (2017). Nitric oxide is essential for the development of aerenchyma in wheat roots under hypoxic stress. Plant Cell Environ..

[146] Munns R. (2008). Tester M Mechanisms of salinity tolerance. Annu. Rev. Plant Biol..

[147] Per T.S., Khan N.A., Reddy P.S., Masood A., Hasanuzzaman M., Khan M.I.R., Anjum N.A. (2017). Approaches in modulating proline metabolism in plants for salt and drought stress tolerance: Phytohormones, mineral nutrients and transgenics. Plant Physiol. Biochem..

[148] Wang H., Liang X., Wan Q., Wang X., Bi Y. (2009). Ethylene and nitric oxide are involved in maintaining ion homeostasis in *Arabidopsis* callus under salt stress. Planta.

[149] Poór P., Tari I. (2011). Ethylene-regulated reactive oxygen species and nitric oxide under salt stress in tomato cell suspension culture. Acta Biol. Szeged..

[150] Poór P., Kovács J., Szopkó D., Tari I. (2013). Ethylene signaling in salt stress-and salicylic acid-induced programmed cell death in tomato suspension cells. Protoplasma.

[151] Poór P., Laskay G., Tari I. (2015). Role of nitric oxide in salt stress-induced programmed cell death and defense mechanisms. Nitric Oxide Action in Abiotic Stress Responses in Plants.

[152] Poór P., Borbély P., Kovács J., Papp A., Szepesi Á., Takács Z., Tari I. (2014). Opposite extremes in ethylene/nitric oxide ratio induce cell death in suspension culture and root apices of tomato exposed to salt stress. Acta Biol. Hung..

[153] Poór P., Kovács J., Borbély P., Takács Z., Szepesi Á., Tari I. (2015). Salt stress-induced production of reactive oxygen-and nitrogen species and cell death in the ethylene receptor mutant Never ripe and wild type tomato roots. Plant Physiol. Biochem..

[154] Lin Y., Yang L., Paul M., Zu Y., Tang Z. (2013). Ethylene promotes germination of Arabidopsis seed under salinity by decreasing reactive oxygen species: Evidence for the involvement of nitric oxide simulated by sodium nitroprusside. Plant Physiol. Biochem..

[155] Sehar Z., Masood A., Khan N.A. (2019). Nitric oxide reverses glucose-mediated photosynthetic repression in wheat (*Triticum aestivum* L.) under salt stress. Environ. Exp. Bot..

[156] Lucena C., Waters B.M., Romera F.J., García M.J., Morales M., Alcántara E., Pérez-Vicente R. (2006). Ethylene could influence ferric reductase, iron transporter, and H^+^-ATPase gene expression by affecting FER (or FER-like) gene activity. J. Exp. Bot..

[157] Romera F.J., García M.J., Alcántara E., PérezVicente R. (2011). Latest findings about the interplay of auxin, ethylene and nitric oxide in the regulation of Fe deficiency responses by Strategy I plants. Plant Signal. Behav..

[158] García M.J., Lucena C., Romera F.J., Alcántara E., Pérez-Vicente R. (2010). Ethylene and nitric oxide involvement in the up-regulation of key genes related to iron acquisition and homeostasis in *Arabidopsis*. J. Exp. Bot..

[159] García M.J., Corpas F.J., Lucena C., Alcántara E., Pérez-Vicente R., Zamarreño Á.M., Bacaicoa E., García-Mina J.M., Bauer P., Romera F.J. (2018). A shoot Fe signaling pathway requiring the OPT3 transporter controls GSNO reductase and ethylene in *Arabidopsis thaliana* roots. Front. Plant Sci..

[160] Zhu X.F., Zhu C.Q., Wang C., Dong X.Y., Shen R.F. (2017). Nitric oxide acts upstream of ethylene in cell wall phosphorus reutilization in phosphorus-deficient rice. J. Exp. Bot..

[161] Liu M., Liu X.X., He X.L., Liu L.J., Wu H., Tang C.X., Zhang Y.S., Jin C.W. (2017). Ethylene and nitric oxide interact to regulate the magnesium deficiency-induced root hair development in *Arabidopsis*. New Phytol..

[162] Jalmi S.K., Bhagat P.K., Verma D., Noryang S., Tayyeba S., Singh K., Sharma D., Sinha A.K. (2018). Traversing the links between heavy metal stress and plant signaling. Front. Plant Sci..

[163] Sahay S., Gupta M. (2017). An update on nitric oxide and its benign role in plant responses under metal stress. Nitric Oxide.

[164] Rodríguez-Serrano M., Romero-Puertas M.C., Zabalza A., Corpas F.J., Gómez M., Del Río L.A., Sandalio L.M. (2006). Cadmium effect on oxidative metabolism of pea (*Pisum sativum* L.) roots. Imaging of reactive oxygen species and nitric oxide accumulation In Vivo. Plant Cell Environ..

[165] Rodríguez-Serrano M., Romero-Puertas M.C., Pazmiño D.M., Testillano P.S., Risueño M.C., Del Río L.A., Sandalio L.M. (2009). Cellular response of pea plants to cadmium toxicity: Cross talk between reactive oxygen species, nitric oxide, and calcium. Plant Physiol..

[166] Chmielowska-Bąk J., Lefèvre I., Lutts S., Deckert J. (2013). Short term signaling responses in roots of young soybean seedlings exposed to cadmium stress. J. Plant Physiol..

